# Automatic no-reference image quality assessment

**DOI:** 10.1186/s40064-016-2768-2

**Published:** 2016-07-16

**Authors:** Hongjun Li, Wei Hu, Zi-neng Xu

**Affiliations:** School of Electronic Information Engineering, Nantong University, Nantong, 226019 People’s Republic of China

**Keywords:** Image quality assessment (IQA), Wavelet domain, Generalized Gaussian density model

## Abstract

No-reference image quality assessment aims to predict the visual quality of distorted images without examining the original image as a reference. Most no-reference image quality metrics which have been already proposed are designed for one or a set of predefined specific distortion types and are unlikely to generalize for evaluating images degraded with other types of distortion. There is a strong need of no-reference image quality assessment methods which are applicable to various distortions. In this paper, the authors proposed a no-reference image quality assessment method based on a natural image statistic model in the wavelet transform domain. A generalized Gaussian density model is employed to summarize the marginal distribution of wavelet coefficients of the test images, so that correlative parameters are needed for the evaluation of image quality. The proposed algorithm is tested on three large-scale benchmark databases. Experimental results demonstrate that the proposed algorithm is easy to implement and computational efficient. Furthermore, our method can be applied to many well-known types of image distortions, and achieves a good quality of prediction performance.

## Background

Objective image quality assessment models typically require the access to a reference image that is assumed to have perfect quality (Wang and Simoncelli 2005; Manap and Shao 2015). In practice, such full-reference methods may not be applicable because the image for reference is not often available (Sheikh et al. 2006). On the other hand, no-reference (NR) or “blind” image quality assessment is an extremely difficult task (Sheikh et al. 2005, 2006; Fang et al. 2015; Leclaire and Moisan 2015; Wang et al. 2015; Lu et al. 2010; Saad et al. 2010; Moorthy and Bovik 2010; Marziliano et al. 2002; Liu et al. 2013; Gao et al. 2013; Du et al. 2004; Xue et al. 2014). The best way to assess the quality of an image is perhaps by visual examination because human eyes are the ultimate receivers in most image processing environments. The subjective quality measures mean opinion score (MOS) has been used for many years. However, the MOS method is inconvenient, slow and expensive for practical use. The goal of objective image and video quality assessment research is to supply a quality metric that can predict perceived image and video quality automatically. Peak signal-to-noise ratio (PSNR) and mean squared error (MSE) are two widely used objective image quality distortion metrics, but they are widely criticized as well, for not correlating well with perceived quality measure. In the past three to four decades, a great deal of efforts have been made to develop a new objective image and video quality measure approaches which incorporate perceptual quality measures by considering human visual system (HVS) characteristics (VQEG 2000).

Most of the image quality assessments require original image as a reference. It will be significant that design a no-reference quality measurement. This topic has attracted a great deal of attention recently, and several methods have been proposed in the literature for objective NR quality assessment. But most NR quality metrics proposed are designed for one or a set of predefined specific distortion type and are unlikely to generalize for evaluating images degraded with other types of distortion. Recently, Sheikh et al. (2005) proposed an NR image quality assessment metric that uses natural scene statistic (NSS) for JPEG2000 compressed images.

The existing state-of-art approach employs the NSS model to do image quality assessment. This is based on the observation that natural images exhibit certain common statistical characters which can be represented by a mathematical model and disturbed by the distorted processing. IQA degree of distortion can be quantized by measuring the change of statistical model. Sheikh’s NSS model metric models the marginal distribution and the joint statistics of wavelet coefficients to evaluate JPEG2000 compressed natural images, which outperforms many other existing methods. However, the experimental results also show that this approach is only applicable to JPEG2000 compressed images. Lu et al. (2010) proposed an NR image quality assessment based on the Sheikh’s IQA and extended to the contourlet domain to solve the inefficiency in directional information capture. Both of them need to obtain the slope of the line which learned from uncompressed natural images in the training set. Because of the parameters are different when computed from the different training sets, the experimental results obtained are also different; it is inconvenient to demonstrate the performance of algorithm by running one time. Some more general NR image quality measures are reported (Saad et al. 2010; Moorthy and Bovik 2010), the blind image quality index (Saad et al. 2010) based on statistics of the Discrete Cosine Transform (DCT) coefficients and with the framework (Moorthy and Bovik 2010) based on natural scene statistics. Despite a promising step towards the general-purpose no-reference quality assessments, the image quality measure heavily relies on a training procedure and their scopes are dependent on the distortions under training. Actually, both of these methods are not real no-reference quality assessments. Therefore, it is necessary to find out a powerful NR quality assessment method.

## Authors contribution

The purpose of this research is to develop an objective NR quality assessment algorithm for natural images. The basic assumption behind natural image statistics-based approach is that most real-world image distortions disturb image statistics and make the distorted image “unnatural”. The distorted measurement based on natural image statistics models can then be used to quantify image quality degradation. Finding a good distortion measures between reference images and distorted images based on some feature set is a challenging task. In particular, here we observe that the marginal distribution of the wavelet coefficients within a given sub-band changes in different ways for different types of image distortions. We then use a generalized Gaussian density model (Portilla et al. 2003) to summarize the marginal distribution of wavelet coefficients, and measure the difference between reference and distortion in order to evaluate image quality. We validate the performance of our algorithm using a wide range of test images and experiments show its superiority. The proposed method performs well in predicting image quality and is easy to implement.

## Methods

### Motivation

The simplest and most widely used full-reference quality metric is the MSE, computed by averaging the squared intensity differences of distorted and reference image pixels, along with the related quantity of PSNR. It is appealing because it is simple to calculate, has clear physical meanings, and mathematically convenient in the context of optimization. But it is not matched perceived visual quality very well (Wang and Bovik 2002). PSNR performs poorly in predicting subjective image quality. To find out a method which can match the visual quality is the main motivation for many researchers in this area.

Statistical modeling is much easier if some process is carried out on the input images. Typical preprocess is done via transformation of image pixel values into a suitable space where simple models with a small number of parameters can describe the data. Wavelet (Chui 1992; Mallet 1999) has recently emerged as an effective tool to provide a perfect theoretical framework and mathematical structures and it have been favored by researchers in many fields. It has been widely used in image enhancement (Heric and Potocnik 2006), image denoising (Li et al. 2010; Li and Suen 2016), segmentation (Kim and Kim 2003), feature extraction (Wee et al. 2008), texture analysis (Do and Vetterli 2002a, b), image compression (Li et al. 2013) and so on (Li and Suen 2016). Image coefficients have many important statistical characters after wavelet transform, such as non-Gaussian marginal distribution, the joint distribution of similarity, intra-scale and inter-scale correlation. Which one can express the image more accurately is an important factor in the establishment. An accurate image statistical model without a reference image can provide a strong theoretical basis.

### Statistical model

One important discovery in the literature of natural image statistics is that the marginal distribution of the coefficients in individual wavelet sub-bands can be well-fitted with a 2-parameter generalized Gaussian density (GGD) model. Mallat proved that the histogram of wavelet coefficients can be fitted by GGD. Define GGD, $p(x;\alpha ,\beta )$,

1 $$p(x;\alpha ,\beta ) = \frac{\beta }{2\alpha \Gamma (1/\beta )}\exp ( - \left| x \right|/\alpha )^{\beta }$$

where,

2 $$\alpha = \sigma \sqrt {\frac{\Gamma (1/\beta )}{\Gamma (3/\beta )}} \quad \beta > 0$$

3 $$\sigma^{2} = \frac{1}{{n^{2} }}\sum\limits_{i,j}^{n} {x_{i,j}^{2} }$$

$\sigma$ is the variance of noise and $\Gamma (.)$ is the Gamma function; Here $\alpha$ models the width of the probability density functions peak, while $\beta$ is inversely proportional to the decreasing rate of the peak. $\alpha$ is referred as the scale parameter while $\beta$ is called the shape parameter. The Gaussian and Laplacian PDFs are special cases in GGD model, when using $\beta$ = 2 and $\beta$ = 1, respectively. The histogram of wavelet coefficients can be fitted by GGD which using the maximum likelihood estimator. As a result, by only two parameters of the GGD, we can capture the marginal distribution of wavelet coefficients in a sub-band accurately. Otherwise, it would require hundreds of parameters by using histogram. This reduces the storage of the image feature significantly, as well as the computational complexity in assessment.

Our purpose then, is to estimate the scale and shape parameters of a reference image in a distorted image. In this paper, it provides a very efficient way to distinguish the coefficient histogram of different types of image distortions. So that only two parameters sequence {α, β} are needed to calculate. Different types of image distortions have different value of parameters. Although GGD model is not characterized well on the wavelet coefficients of the distorted image, the parameters of model are important factors to calculate the distortion. To prove this opinion, we analyzed the relation of parameters between reference image and distorted image.

**Fig. 1:**
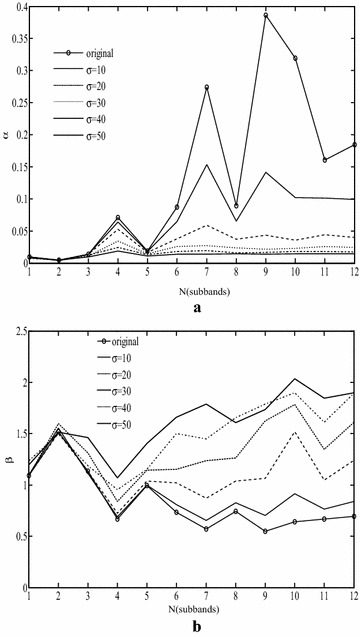
The parameters of GGD change in different scale under write Gaussian noise. **a** α Parameter, **b** β parameter

Figure 1 shows the changes of the parameters of GGD as a function of sub-band number for different sigma values. Finer sub-bands coefficients and coarser sub-bands coefficients are uncorrelated, they are still statistically dependent. Furthermore, this dependency cannot be eliminated through further linear transformation. The structure of the relationship between finer sub-bands coefficients and coarser sub-bands coefficients become more apparent upon transforming to the log domain (Buccigrossi and Simoncelli 1999). In the following section we will analyze these two parameters of GGD in detail, and discuss the character of them when they change in different distorted images.

**Fig. 2:**
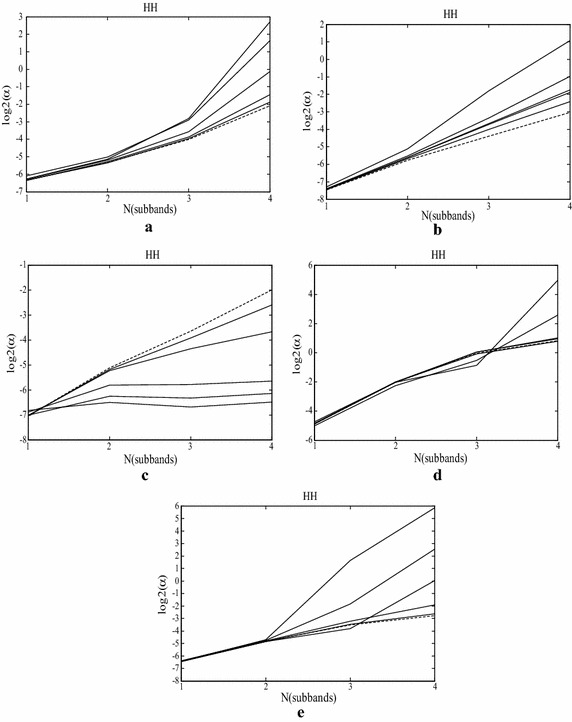
Log_2_ (α) for image at different type of distorted image in diagonal sub-band. **a** JPEG2000 compressed image; **b** Gaussian blurred image; **c** white Gaussian noise contaminated; **d** JPEG compressed image; **e** fast-fade

It is an interesting observation that when the means of (log_2_ (α)) are plotted in an enumeration of the sub-bands, the plot is approximately linear. Figure 2 depicts the value of (log_2_ (α)) for different images under different distort degrees (solid curves) and their corresponding reference images (dash curve) in diagonal sub-bands.

Figure 2 shows the value of scale parameter in log domain of different distortions. The values of (log_2_ (α)) in the diagonal sub-bands of reference image are quite close to linear, While the values of (log_2_ (α)) in the diagonal sub-band of distorted images is close to the reference image in the low sub-bands. For JPEG2000, JPEG, Gaussian-blur and Fast-fade images, the value of (log_2_ (α)) changes approximately in the same way. To the white Gaussian noise image, the value of (log_2_ (α)) is not very close to the description above, especially when the noise is in high density, this is shown in Fig. 2c.

To make the method adapt to the images distorted by Gaussian noise well, we used the deviated value of scale parameters in each sub-band to improve the accuracy of image quality assessment method. Deviated value is defined as,

4 $$D_{i} = \log_{2} (\alpha_{i + 1} ) - \log_{2} (\alpha_{1} )\quad i = 1, \ldots ,(scale - 1)$$

**Fig. 3:**
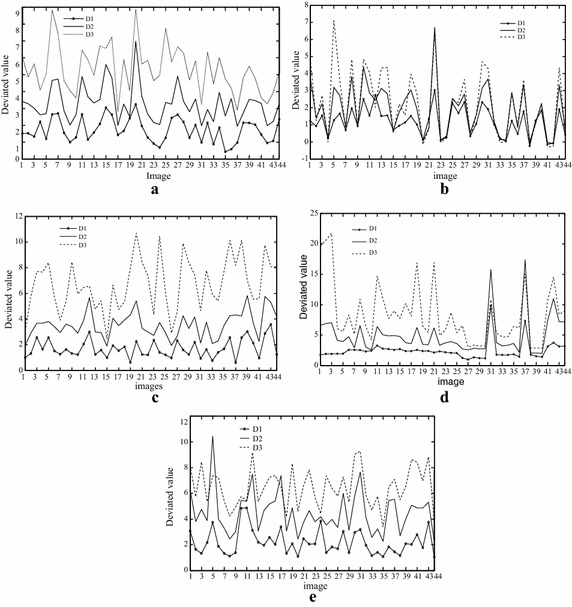
The deviated value of scale parameter in images of different distortions. **a** JPGE, **b** white Gaussian noise, **c** JPGE2000, **d** fast fade, **e** Gaussian blur

We calculate the deviated value (D) of some images in the LIVE database II (http://live.ece.utexas.edu/research/quality); the result is showed in Fig. 3. The images distorted by Gaussian noise are not satisfying the inequality $0 < D1 < (D2,D3)$. So we can use this character to sort distorted images into two kinds, with and without Gaussian noise.

**Fig. 4:**
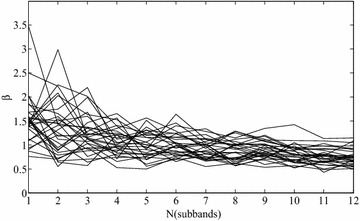
The value of β in different scales and directions on natural images

It is of interest to know the common range for the values of scale parameters in GGDs for natural images. For typical natural images which are dominated by smooth regions, the values for β are found between 0.5 and 1. Figure 4 shows the histogram of the estimated values of β from reference images in LIVE database II in four decomposition scales. Most of estimated β in finer scales are between 0.5 and 1 in finer scales.

It is difficult to obtain the value β of reference image accurately from the distorted image. We search the optimal β as a value of reference image from different distorted images. In Table 1 we show the different values of shape parameters in the [0.5 1] and these different values may have different results of quality assessments. Numerous experiments show that when β = 0.7 obtains a good quality assessment in most distorted images, expect Gaussian noise. Therefore, before assess the test image, we first use the scale parameters to sort the test image into two kinds, and then choose the shape parameter in different way. Without the Gaussian noise distortion we set β = 0.7, otherwise set it as a small value in search area.

**Table 1:** Performance evaluation of image quality measures in different value of $\beta_{e}$

$\beta_{e}$	0.5	0.6	0.7	0.8	0.9	1.0
JPEG2000 (JPEG2k)						
CC	0.61	0.79	*0.88*	0.83	0.82	0.80
RMS	13.2	11.3	*8.6*	9.4	10.5	11.0
ROCC	0.8	0.82	*0.94*	0.92	0.90	0.85
JPEG (JPEG)						
CC	0.59	0.75	*0.90*	0.88	0.79	0.6
RMS	14	13.6	*11.5*	13.3	13.4	13.9
ROCC	0.71	0.74	*0.90*	0.84	0.75	0.72
White Gaussian noise (WGN)						
CC	*0.95*	0.91	*0.88*	0.84	0.75	0.65
RMS	*5.1*	7.8	*8.9*	10.0	11.7	12.8
ROCC	*0.97*	0.95	*0.93*	0.89	0.84	0.77
Gaussian blur (GB)						
CC	0.78	0.8	*0.90*	0.82	0.84	0.85
RMS	9.9	9.7	*8.7*	9.4	8.8	8.5
ROCC	0.77	0.85	*0.97*	0.9	0.91	0.91
Fast fading (FF)						
CC	0.59	0.65	*0.85*	0.75	0.73	0.68
RMS	12.6	12	*10.5*	10.9	11.3	11.3
ROCC	0.81	0.86	*0.91*	0.88	0.85	0.85

Italic values indicate the best performance

### Distortion measure

Let p(x) and q(x) denote the probability density functions of wavelet coefficients in the same sub-band of two images. Let x = {x_1_,…,x_N_} be a set of N randomly and independently selected coefficients. The likelihoods of x being drawn from p(x) and q(x) are

5 $$l(p|x) = \frac{1}{N}\sum\limits_{n = 1}^{N} {\log p(x_{n} )}$$

6 $$l(q|x) = \frac{1}{N}\sum\limits_{n = 1}^{N} {\log q(x_{n} )}$$

respectively.

The difference of the likelihoods between p(x) and q(x) is

7 $$l(p|x) - l(q|x) = \frac{1}{N}\sum\limits_{n = 1}^{N} {\log \frac{{p(x_{n} )}}{{q(x_{n} )}}}$$

In previous work, a number of authors have pointed out that relationship between Kullback–Leibler distance (KLD) and likelihoods function, and used KLD to compare images, mainly for classification and retrieval purposes. We obtain the following form for the KLD between two GGDs as

8 $$D(p(.,\alpha_{1} ,\beta_{1} )||q(.,\alpha_{2} ,\beta_{2} )) = \log \left( {\frac{{\beta_{1} \alpha_{2} \Gamma (1/\beta_{2} )}}{{\beta_{2} \alpha_{1} \Gamma (1/\beta_{1} )}}} \right) + \left( {\frac{{\alpha_{1} }}{{\alpha_{2} }}} \right)^{{\beta_{2} }} \frac{{\Gamma ((\beta_{2} + 1)/\beta {}_{1})}}{{\Gamma (1/\beta {}_{1})}} - \beta {}_{1}$$

Therefore, the KLD can be estimated very effectively by using the model parameters. The distance function defined in Eq. (8) is a function of three variables: the ratio of two scale parameters α_1/_α_2_ and two shape parameters β_1_ and β_2_. In this paper, we only selected the diagonal sub-bands to reduce the complexity of this method. Wavelet coefficients in each sub-band can be used to calculate two model parameters {α, β} and KLD. The value of KLD is small when the parameters {α_1_, β_1_} and {α_2_, β_2_} are close, which is true for typical natural images. So we used KLD to obtain the difference of coefficient distributions between a distorted image and reference image.

## Implementation

### Estimate GGD parameters of reference image from distorted image

In this paper, we attempt to design an efficient feature extraction method with less computation complexity. The feature is calculated in each diagonal sub-bands and then estimated by the GGD model. We observe from the experiments that the sub-band statistic result is different in different scales and orientations. As the degree of distortion increases, the parameters of GGD in finer sub-bands increase faster than the parameters in coarser sub-bands. It is interesting that parameters of GGD in the coarser diagonal sub-bands are plotted approximately linear, and very close to the uncompressed natural images. Figure 2 shows each type of distorted natural images. Hence, from the coarser diagonal sub-bands, one could predict the slope of line and assume that reference image have the same slope. This line yields the estimating the value of (log_2_ (α)) in finer diagonal sub-bands under the reference image, and the distorted image quality can be evaluated by the derivation of scale parameters. This is shown in Fig. 2 as well, where the (log_2_ (α)) is plotted for an image in different types distorted image. The value of (log_2_ (α)) in distorted image (show in solid curves) is quite close to the reference image (dashed curve) in coarser diagonal sub-band.

The slope of the line can be calculated by (log_2_ (α)) in coarser diagonal sub-bands, while the offsets in other diagonal sub-bands can be calculated by the difference between $dc$ and $dp$, defined as:

9 $$dc_{i} = \{ \log (\alpha_{i} )|i = 3 \ldots (scale)\}$$

10 $$dp_{i} = \{ (\log_{2} (\alpha_{2} ) - \log_{2} (\alpha_{1} )) \times i + (2\log_{2} (\alpha_{1} ) - \log_{2} (\alpha_{2} ))|i = 3 \ldots (scale)\}$$

where $i$ is decomposition scale, $\alpha_{i}$ is the value of the parameters of GGD in diagonal sub-bands in a distorted image.$dc_{i}$ can be obtained from the parameters of GGD and $dp_{i}$ is a calculated value by the parameters of GGD in diagonal sub-bands of scale $i$ which behaviors the value of a natural image. So the GGD parameters in a distorted image and a reference image can be obtained by the method above, then we use these parameters to calculate the distortion between the distorted image and reference image.

11 $$d_{i} = (p(.,\alpha_{ci} ,\beta_{ci} )||q(.,\alpha_{ei} ,\beta_{ei} ))\text{ }i \in \{ 3, \ldots (scale)\}$$

where $\alpha_{ci} ,\beta_{ci}$ are the parameters computed by GGD on the histogram of coefficients. $\alpha_{ei}$ is calculated by the slope of the line in Eq. (10). $\beta_{e}$ is a value of reference image about different distorted images.

For typical natural images which are dominated by smooth regions, the values of β are found between 0.5 and 1. In Table 1 we show the result of image quality assessment with different values of $\beta_{e}$ in the [0.5 1]. We find that when $\beta_{e}$ = 0.7, it makes a good performance in most distortions. Since it is difficult to estimate an accurate value of shape parameter in reference image from different distorted images, so it is a good choice to set $\beta_{e}$ as a fixed value, especially when input a single image without any training set.

$d_{i}$ is calculated by Eq. (11), So the overall distortion between the distorted image and reference image is defined as:

12 $$Q = \log_{2} \left( {1 + \lambda \sum\limits_{i = 3}^{s} {d_{i} } } \right)$$

where $s$ is decomposition scales, $d_{i}$ is the estimation of the KLD between distorted image and reference image, $\lambda$ is a constant used to control the scale of the distortion measure.

### Quality assessment method

**Fig. 5:**
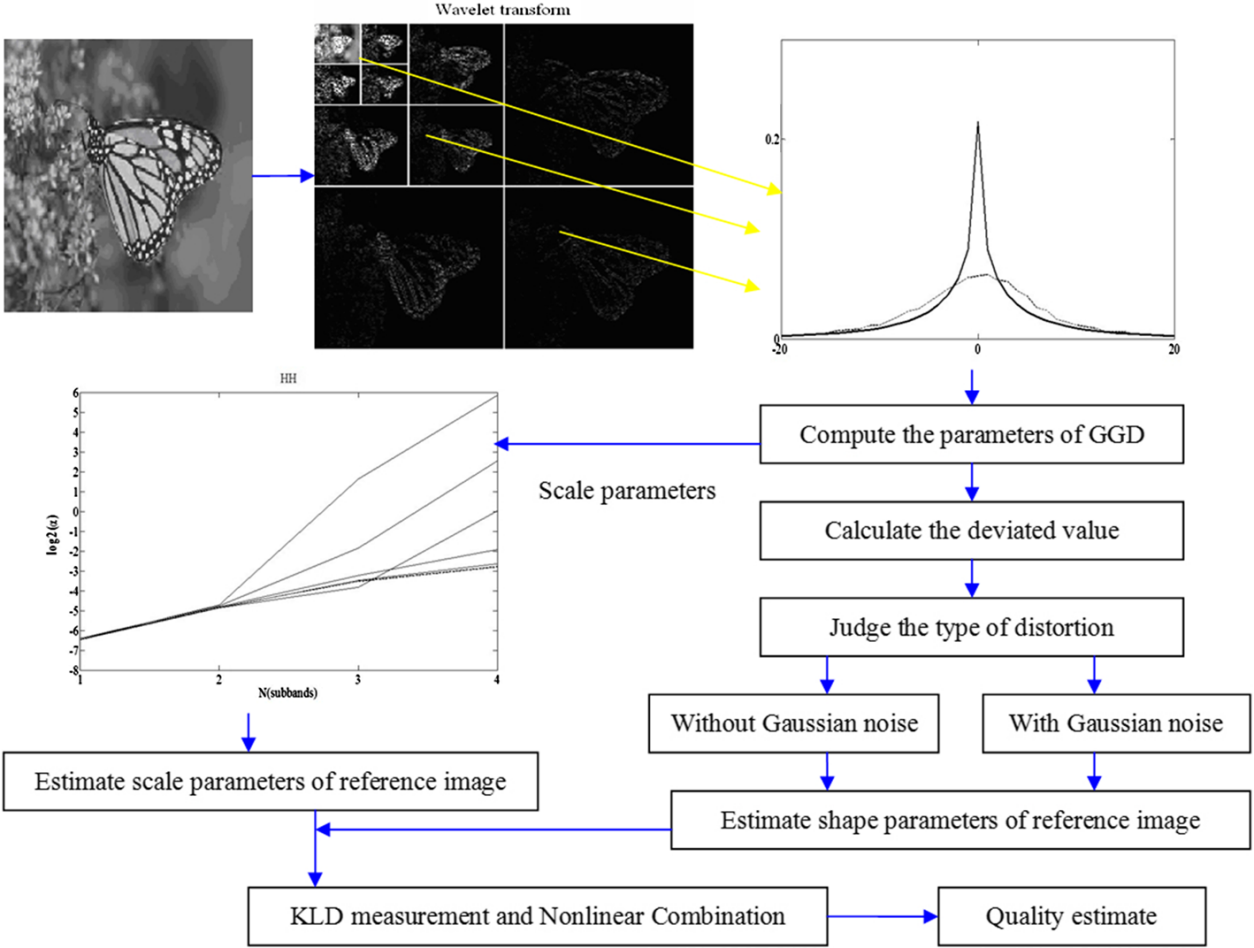
Quality analysis systems

The quality analysis system for the distorted images is illustrated in Fig. 5. The biorthogonal 9/7 wavelet with four levels of decomposition is used to transform. For each selected sub-band, the histogram of the coefficients is computed and its feature parameters are then estimated using GGD model. The major purpose of selecting a diagonal subset of all sub-bands is to reduce the algorithm complexity.

## Test

We use the LIVE database II to evaluate the performance of the method we proposed. The database contains 29 high-resolution original images altered with five types of distortion at different levels. The distorted images were divided into five datasets. JPEG2000 compressed images (227 images); JPEG compressed images (233 images); and White Gaussian Noise (WGN), Gaussian Blur (GB), Fast Fading (FF) each containing 174 images, respectively. Subjects were asked to provide their perception of quality on a continuous linear scale that was divided into five equal regions marked with “Bad”, “Poor”, “Fair”, “Good” and “Excellent”, respectively. The raw scores for each subject were converted into Z-scores and rescaled within each dataset to fill the range from 1 to 100. MOS and the standard deviation between subjective scores were then computed for each image.

**Fig. 6:**
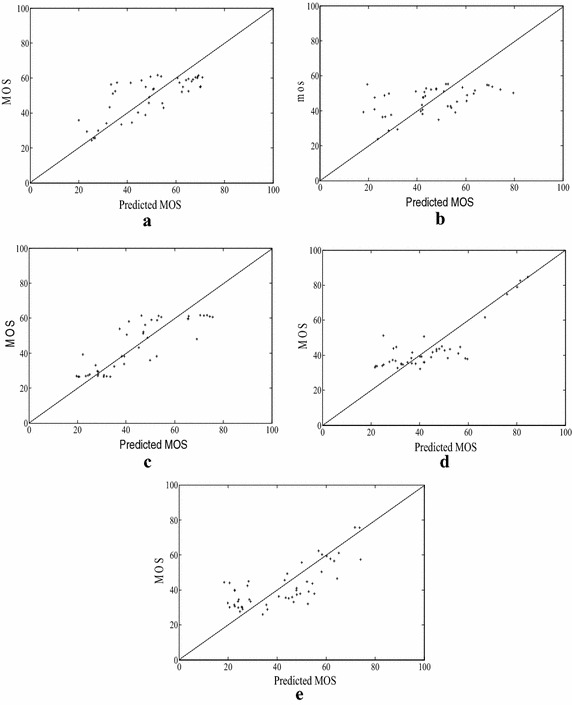
Scatter plots of mean opinion score (MOS) versus model prediction in the LIVE database. **a** JPEG2000, **b** JPEG, **c** white Gaussian noise, **d** Gaussian blur, **e** fast fading

The proposed method used KLD value to quantify the performance of the proposed quality assessment method. We used the four parameters logistic functions to provide a nonlinear mapping between objective and subjective scores proposed in video quality experts group (VQEG). Figure 6 shows the scatter plots (in which each data point represents one test image) of true mean opinion score versus the predicted score by the method proposed. After such a nonlinear mapping, the correlation coefficients between the predicted and true subjective scores are calculated to evaluate prediction accuracy and the spearman rank-order correlation coefficient is computed to evaluate prediction monotonicity.

The performance evaluation results are shown in Table 2. It can be seen that our method performs quite well for a wide range of distortion types. Specifically, it provides better prediction accuracy (higher correlation coefficients), better prediction monotonicity (higher Spearman rank-order correlation coefficients) and better prediction efficient(Root Mean Square). Besides, our method needs no additional information from training sets; it is the main outstanding of our method which is better than papers (Sheikh et al. 2005; Lu et al. 2010). In most images except JPEG, the method we proposed is better than blind image quality indices (BIQI) (Moorthy and Bovik 2010) and nearly to the reference image quality assessment such as PSNR, structural similarity (SSIM). We also compare the state-of-the-art blind image quality assessment (BIQA) models proposed in Ref. Xue et al. (2014). Our proposed method is very closely its performance. Therefore, we believe our method is a reasonable and useful choice in practical quality assessment systems.

**Table 2:** Performance evaluation of image quality measures using the LIVE database

Dataset	JPEG2k	JPEG	WGN	GB	FF
Correlation coefficient					
PSNR	0.85	0.87	0.92	0.74	0.85
SSIM	0.94	0.94	0.98	0.90	0.95
Ref. (Sheikh et al. 2005)	0.92	0.38	0.93	0.76	0.71
Ref. (Lu et al. 2010)	0.84	0.58	0.95	0.85	0.84
BIQI (Moorthy and Bovik 2010)	0.80	0.90	0.95	0.82	0.73
BIQA (Xue et al. 2014)	0.87	0.94	0.95	0.89	0.85
Proposed method	0.88	0.90	0.95	0.90	0.85
Rank order correlation coefficient					
PSNR	0.85	0.87	0.93	0.72	0.85
SSIM	0.93	0.94	0.96	0.90	0.94
Ref. (Sheikh et al. 2005)	0.90	0.27	0.91	0.70	0.71
Ref. (Lu et al. 2010)	0.82	0.57	0.63	0.85	0.82
BIQI (Moorthy and Bovik 2010)	0.79	0.89	0.95	0.84	0.70
BIQA (Xue et al. 2014)	0.93	0.95	0.98	0.94	0.90
Proposed method	0.94	0.90	0.97	0.97	0.91
Root mean square					
PSNR	12.8	14.8	10.7	12.2	14.5
SSIM	8.6	10.1	5.2	8.0	8.5
Ref. (Sheikh et al. 2005)	10.3	30.2	10.7	13.0	19.3
Ref. (Lu et al. 2010)	8.7	24.4	9.62	8.4	14.8
BIQI (Moorthy and Bovik 2010)	14.9	13.8	8.4	10.3	19.3
BIQA (Xue et al. 2014)	9.7	10.0	4.9	9.0	10.0
Proposed method	9.4	11.5	5.1	8.7	10.5

To test the generalization ability of the proposed model with respect to distortion types, we conducted further experiments on the entire TID2008 (Ponomarenko et al. 2009) and CISQ (Larson and Chandler 2010). The TID2008 database contains 25 reference images and 1700 distorted images (25 reference images × 17 types of distortions × 4 levels of distortions). The CISQ database consists of 30 original images and their distorted counterparts with six types of distorted at four to five different levels of distortion. For the CISQ and TID2008 databases, we mainly consider the 4 common types of distortion that appear in the LIVE database II, i.e., JPEG2000, JPEG, WGN, and GB. We evaluated several state-of-the-art algorithms, i.e., BIQI (Moorthy and Bovik 2010) and BIQA (Xue et al. 2014). The results are listed in Table 3. The proposed method shows clear advantage over the BIQI, and similar to the state-of-the-art BIQA method.

**Table 3:** Performance evaluation of image quality measure

Rank order correlation coefficient	BIQI (Moorthy and Bovik 2010)	BIQA (Xue et al. 2014)	Proposed method
CSIQ			
WGN	0.62	*0.94*	0.92
JPEG	0.84	0.93	*0.94*
JPEG2k	0.76	*0.91*	0.89
GB	0.81	0.90	*0.92*
TID2008			
WGN	0.55	*0.90*	0.88
GB	0.89	0.88	*0.92*
JPEG	0.90	*0.93*	0.90
JPEG2k	0.81	*0.92*	0.91

Italic values indicate the best performance

The computational complexity is also an important factor when evaluating image quality assessments. The complexity of proposed method is similar to BIQI (Moorthy and Bovik 2010), the computational complexity is $O(N)$, where $N$ is the total number of image pixels. As a rough comparison, the execution time for processing an image is about 0.5 s using MATLAB implementation.

## Conclusion

In this paper, an improved image assessment model is proposed to do image quality assessment without any reference. The image is decomposed by wavelet into multi-scale and multi-directional sub-bands. The authors use the Kullback–Leibler distance on the marginal probability distribution of wavelet coefficients in distorted image as a measure of image distortion. A generalized Gaussian model is employed to summarize the marginal distribution of wavelet coefficients of images, so that only a relatively small number of features are needed for the evaluation of image quality. The dependencies are captured by the parameters of GGD to estimate the corresponding value in the reference image. The method proposed is easy to implement and efficient in computation.

The experiments on numerous images from database demonstrate the efficiency of the method we proposed. Several properties of the method we proposed may be of interest for real-world users. First, it is a general-purpose method that performs well for a wide range of distortion types and need no information of reference image. Second, the method is easy to implement, computational efficient, and need less parameters. Third, since the measurement is based on marginal distributions of wavelet coefficients, the method is insensitive to small geometric distortions such as spatial translation, rotation and scaling. In the future, the method may be further improved by incorporating joint statistics of wavelet coefficients, which are much more powerful in characterizing the statistical structures of natural images.

## References

[CR1] Buccigrossi RW, Simoncelli EP (1999). Image compression via joint statistical characterization in the wavelet domain. IEEE Trans Image Process.

[CR2] Chui CK (1992). An Introduction to wavelet.

[CR3] Do MN, Vetterli M (2002). Rotation invariant texture characterization and retrieval using steerable wavelet-domain hidden Markov models. IEEE Trans Multimed.

[CR4] Do MN, Vetterli M (2002). Wavelet-based texture retrieval using generalized Gaussian density and Kullback–Leibler distance. IEEE Trans Image Process.

[CR5] Du J, Yu Y, Xie S (2004). NR objective quality assessment of digital image/video: a neural-network approach. J Inf Comput Sci.

[CR6] Fang Y, Ma K, Wang Z (2015). No-reference quality assessment of contrast-distorted images based on natural scene statistics. IEEE Signal Process Lett.

[CR7] Gao X, Gao F, Tao D, Li X (2013). Universal blind image quality assessment metrics via natural scene statistics and multiple kernel learning. IEEE Trans Neural Netw Learn Syst.

[CR8] Heric D, Potocnik B (2006). Image enhancement by using directional wavelet transform. J Comput Inf Technol.

[CR9] Kim J, Kim H (2003). Multiresolution-based watersheds for efficient image segmentation. Pattern Recogn Lett.

[CR10] Larson EC, Chandler DM (2010). Most apparent distortion: full-reference image quality assessment and the role of strategy. J Electr Imaging.

[CR11] Leclaire A, Moisan L (2015). No-reference image quality assessment and blind deblurring with sharpness metrics exploiting fourier phase information. J Math Imaging Vis.

[CR12] Li Hongjun, Suen Ching Y (2016). A novel non-local means image denoising method based on grey theory. Pattern Recogn.

[CR13] Li H, Suen CY (2016). Robust face recognition based on dynamic rank representation. Pattern Recogn.

[CR14] Li H-J, Zhao Z-M, Yu X-L (2010). A novel image denoising algorithm in wavelet domain using total variation and grey theory. Eng Comput.

[CR15] Li HJ, Xie ZG, Hu W (2013) An image compression method using sparse representation and grey relation. In: 2013 IEEE international conference on grey systems and intelligent service, pp 53–56

[CR16] Liu TJ, Lin W, Kuo C-CJ (2013). Image quality assessment using multi-method fusion. IEEE Trans Image Process.

[CR17] Lu W, Zeng K, Tao D, Yuan Y, Gao X (2010). No-reference image quality assessment in contourlet domain. Neurocomputing.

[CR18] Mallet S (1999). A wavelet tour of signal processing.

[CR19] Manap RA, Shao L (2015). Non-distortion-specific no-reference image quality assessment: a survey. Inf Sci.

[CR20] Marziliano P, Dufaux F, Winkler S (2002). A no-reference perceptual blur metric. Int Conf Image Process.

[CR21] Moorthy AK, Bovik AC (2010). A two-step framework for constructing blind image quality indices. IEEE Signal Process Lett.

[CR22] Ponomarenko N, Lukin V, Zelensky A, Egiazarian K, Carli M, Battisti F (2009). TID2008—a database for evaluation of full-reference visual quality assessment metrics. Adv Mod Radioelectron.

[CR23] Portilla J, Strela V, Wainwright MJ, Simoncelli EP (2003). Image denoising using scale mixture of Gaussian in the wavelet domain. IEEE Trans Image Process.

[CR24] Saad MA, Bovik AC, Charrier C (2010). A DCT statistics-based blind image quality index. IEEE Signal Process Lett.

[CR25] Sheikh HR, Bovik AC, Cormack L (2005). No-reference quality assessment using natural scene statistics: JPEG 2000. IEEE Trans Image Process.

[CR26] Sheikh HR, Sabir MF, Bovik AC (2006). A statistical evaluation of recent full reference image quality assessment algorithms. IEEE Trans Image Process.

[CR33] Sheikh HR, Wang Z, Cormack et al. LIVE image quality assessment database release 2. http://live.ece.utexas.edu/research/quality

[CR27] VQEG (2000) Final report from the video quality experts group on the validation of objective models of video quality assessment. http://www.vqeg.org/

[CR28] Wang Z, Bovik AC (2002). A universal image quality index. IEEE Signal Process Lett.

[CR29] Wang Z, Simoncelli EP (2005). Reduced-reference image quality assessment image statistic model. Proc SPIE Hum Vis Electron Imaging.

[CR30] Wang C, Shen M, Yao C (2015). No-reference quality assessment for DCT-based compressed image. J Vis Commun Image Represent.

[CR31] Wee A, Grayden D, Zhu Y, Petkovic-Duran K, Smith D (2008). A continuous wavelet transform algorithm for peak detection. Electrophoresis.

[CR32] Xue W, Mou X, Zhang L, Bovik A, Feng X (2014). Blind image quality assessment using joint statistics of gradient magnitude and laplacian features. IEEE Trans Image Process.

